# Early Functional and Radiological Outcomes of Radial Head Replacement in the Management of Radial Head Fractures: A Prospective Study

**DOI:** 10.7759/cureus.98121

**Published:** 2025-11-29

**Authors:** Maninder Singh, Rajesh Kapila, Sk Julfikar Hossain

**Affiliations:** 1 Orthopedics and Traumatology, Government Medical College, Amritsar, IND

**Keywords:** elbow fractures, mason type iii/iv fractures, radial head, radial head arthroplasty, radial head fractures, replacement

## Abstract

Background

Radial head fractures, particularly Mason type III and IV, often require radial head replacement (RHR) to restore elbow stability and function when fractures are unreconstructable. This study evaluates the short-term functional and radiological outcomes of RHR in managing complex radial head fractures

Methods

A prospective study was conducted from January 2023 to January 2025 at Government Medical College, Amritsar, India, involving 25 patients with Mason type III (n=21, 84%) and type IV (n=4, 16%) radial head fractures treated with monopolar cemented RHR. Functional outcomes were assessed using the Mayo Elbow Performance Score (MEPS) at 6, 12, and 24 weeks post-surgery, while radiological outcomes evaluated prosthesis stability, alignment, and complications. Statistical analysis included Fisher's exact test for categorical variables and t-tests/ANOVA for continuous variables (p<0.05).

Results

The mean age was 40.2 ± 13.5 years (16, 64% male). The mean MEPS improved from 76.2 ± 12.2 at six weeks to 87.8 ± 12.1 at 24 weeks (p<0.001), with 11 (44%) achieving excellent (MEPS ≥90) and 12 (48%) good (MEPS 75-89) outcomes. Stable prosthesis alignment was achieved in all patients by 12 weeks (mean 8.1 ± 1.4 weeks). Complications occurred in eight (32%), including infection in two (8%) and stiff elbow in two (8%), with no significant association with Mason type (p=0.275). No significant difference in MEPS was observed between Mason type III and IV fractures (p=0.523).

Conclusion

RHR is effective for Mason type III and IV radial head fractures, yielding good functional outcomes and reliable prosthesis stability with a notable complication rate. Addressing associated injuries is crucial for optimizing outcomes. Larger studies with longer follow-up are needed to assess long-term prosthetic durability.

## Introduction

Radial head fractures account for approximately one-third of all elbow fractures, with an incidence of 2.5-2.9 per 10,000 individuals annually [1]. These injuries typically result from high-energy trauma, such as road traffic accidents or falls on an outstretched hand [2,3]. The radial head is critical for elbow stability, acting as a secondary valgus stabilizer, particularly when the coronoid process is compromised [4]. Non-displaced fractures (Mason type I) are managed conservatively, but displaced or comminuted fractures (Mason types II-IV) often require surgical intervention [5].

Surgical options include open reduction and internal fixation (ORIF), radial head excision, or radial head replacement (RHR). ORIF is suitable for reconstructable fractures, but comminuted or unstable fractures (Mason types III and IV) often necessitate RHR to restore elbow stability and prevent complications like valgus instability or proximal radial migration [6,7]. Radial head excision is now limited to low-demand patients due to risks of long-term instability [8]. Advances in prosthetic design have improved RHR outcomes, making it a preferred option for complex fractures [9].

This prospective study evaluates the short-term functional and radiological outcomes of radial head replacement in patients with Mason type III and IV radial head fractures, assessing elbow function, prosthesis stability, and complications over a six-month follow-up period.

## Materials and methods

This prospective study was conducted at the Department of Orthopedics, Government Medical College, Amritsar, from January 2023 to January 2025. A total of 25 patients with radial head fractures presenting to the outpatient or emergency department were enrolled. The study was initiated after obtaining departmental ethical clearance, and formal institutional ethical approval was subsequently granted in 2024, prior to data analysis. Informed written consent was secured from all participants.

The study included patients of both sexes, aged more than 18 years, who had Mason type III and IV radial head fractures, and without major comorbid conditions precluding surgery. Patients with associated ligamentous injuries or elbow dislocation requiring radial head replacement were also included. The exclusion criteria included patients aged less than 18 years, Mason type I and II radial head fractures, with compound fractures, concomitant neurovascular injuries, medically unfit patients, and patients unwilling to provide consent for surgery.

**Figure 1:**
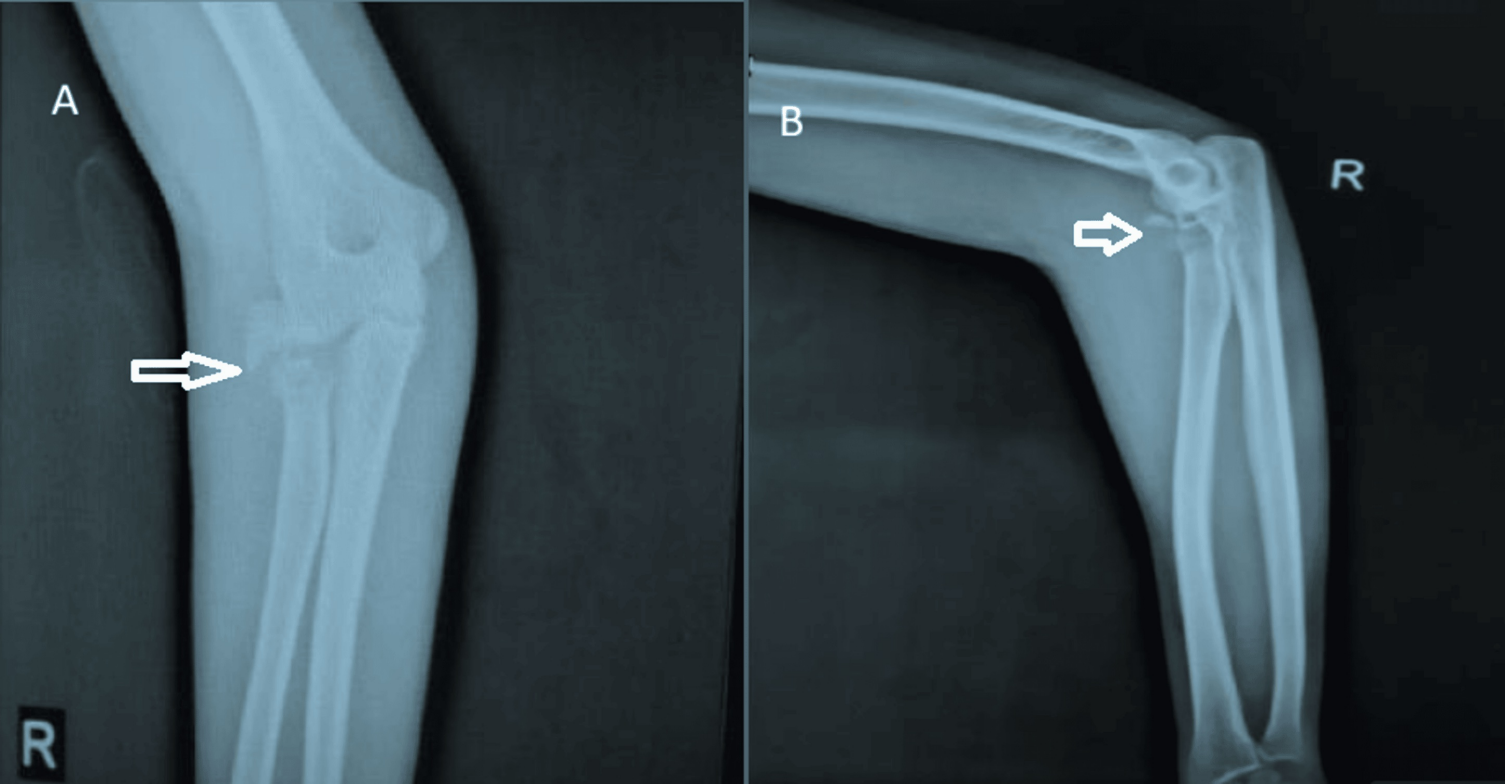
A) Preoperative radiograph showing the anteroposterior (AP) view of the right elbow with Mason type III radial head fracture. B) Preoperative radiograph showing the lateral view of the right elbow with Mason type III radial head fracture.

**Figure 2:**
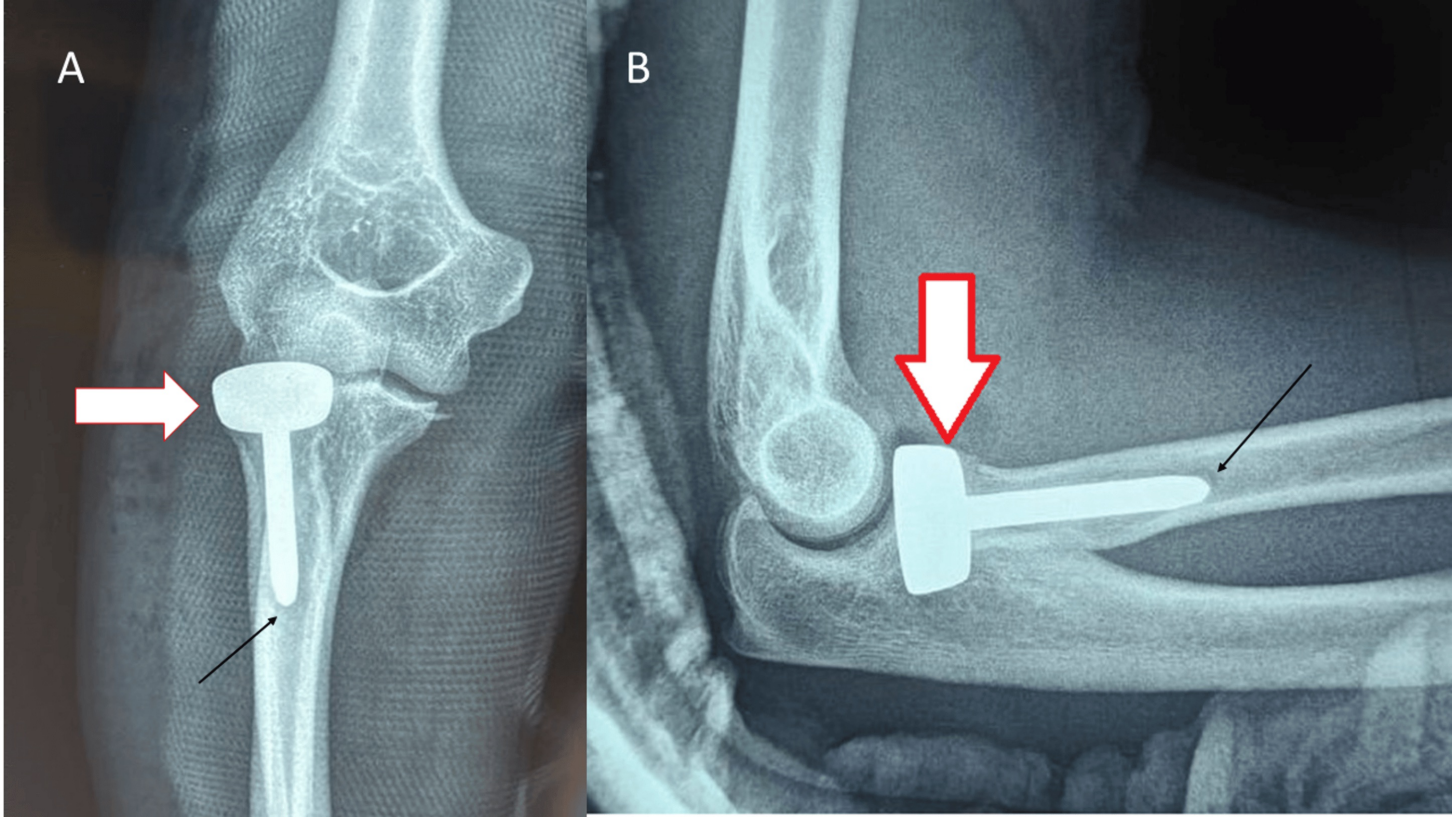
A) Post-operative radiograph showing the anteroposterior (AP) view of the right elbow with Mason type III radial head fracture. B) Post-operative radiograph showing the lateral view of the right elbow with Mason type III radial head fracture. The white arrow indicates the radial head prosthesis, while the black arrow highlights the cement mantle surrounding the implant.

Before surgery, a detailed history was obtained, a comprehensive clinical examination was done, routine investigations were sent, and the patient was investigated with radiographs, a CT scan if required, and MRI if ligamentous injury was suspected. Radial head replacement was performed under general or regional anesthesia. The Kocher approach was used in most cases for exposure of the radial head. The Kaplan approach was used in cases with associated lateral ligament injury, and the Boyd approach was reserved for cases with associated olecranon fractures. All surgeries were performed by a single senior surgeon. Postoperative rehabilitation and assessment protocols were standardized for all patients to minimize bias related to approach selection. The fractured radial head and neck were excised, and the medullary canal was prepared with a rasp. Palacos R bone cement (Wehrheim, Heraeus Medical, Germany) was injected into the medullary canal with a cement restrictor to prevent distal extrusion, and a monopolar radial head prosthesis (Siora Surgicals Pvt. Ltd, Delhi, India) was implanted in all cases. Intraoperative alignment and sizing were verified using trial heads and fluoroscopy to ensure accurate radio-capitellar congruence and to avoid overstuffing (Figures 1, 2). The annular ligament and soft tissues were repaired, and the wound was closed in layers. Postoperatively, a posterior above-elbow plaster slab was applied for approximately two weeks to provide pain relief and soft tissue rest. After slab removal, active and passive range of motion exercises were initiated under supervision to prevent stiffness and promote early functional recovery.

Patients were followed up at six weeks, 12 weeks, and six months post-surgery. Clinical outcomes were assessed using the Mayo Elbow Performance Score (MEPS), as described by Morrey et al. in 1993, evaluating pain, motion, stability, and function (maximum score: 100) [8]. Radiological assessments included X-rays to evaluate radio-capitellar congruence, heterotopic ossification, implant loosening, and overstuffing. The time to achieve a stable prosthesis (defined as maintenance of the prosthesis position without evidence of migration, tilting, or loosening on serial radiographs, along with radiographic signs of fracture union) was recorded. Complications were noted.

Data were compiled in Excel (Microsoft, Redmond, Washington) and analyzed using SPSS for Windows version 24.0 (IBM Inc., Armonk, New York). Descriptive statistics were used for demographic and outcome measures. Categorical variables (e.g., complications by Mason type) were analyzed using Fisher's exact test due to low expected frequencies, while continuous variables (e.g., MEPS) were compared using independent t-tests or ANOVA, with a p-value <0.05 considered statistically significant. Within-group comparisons of Mayo Elbow Performance Scores (MEPS) at different follow-up intervals (six, 12, and 24 weeks) were analyzed using a paired t-test, while between-group differences were evaluated using independent t-tests or ANOVA as appropriate.

## Results

The study included 25 patients with Mason type III (n=21, 84%) and type IV (n=4, 16%) radial head fractures. The mean age was 40.2 ± 13.5 years, with 16 (64%) male and nine (36%) female patients. The right elbow was affected in 14 (56%) of cases, and the left in 11 (44%). The modes of injury were roadside accidents (21, 84%), falls (3, 12%), and physical assault (n=1, 4%). Associated injuries were present in nine (36%) of cases, including olecranon fractures (n=3, 12%), elbow dislocations (n=3, 12%), coronoid fracture (n=1, 4%), Essex-Lopresti injury (1, 4%), and terrible triad of the elbow (n=1, 4%) (Table 1).

**Table 1:** Demographics of the study population

Category	Subcategory	Number of patients	Percentage (%)
Sex distribution	Male	16	64.00%
	Female	9	36.00%
Side affected	Right	14	56.00%
	Left	11	44.00%
Mode of injury	Roadside accident	21	84.00%
	Fall	3	12.00%
	Physical assault	1	4.00%
Mason classification	Type III	21	84.00%
	Type IV	4	16.00%

The mean MEPS improved significantly from 76.2 ± 12.2 at six weeks to 87.8 ± 12.1 at 24 weeks (p<0.001, paired t-test). At 24 weeks, 11 (44%) of patients achieved excellent outcomes (MEPS ≥90), 12 (48%) had good outcomes (MEPS 75-89), one (4%) had fair outcomes (MEPS 60-74), and one (4%) had poor outcomes (MEPS <60). Stable prosthesis alignment was achieved in all patients by 12 weeks, with a mean time of 8.1 ± 1.4 weeks (Table 2).

**Table 2:** Descriptive statistics of outcome measures MEPS - Mayo Elbow Performance Score

Outcome measure	Mean ± SD (n=25)
MEPS (6th week)	76.2 ± 12.2
MEPS (12th week)	85.2 ± 13.0
MEPS (24th week)	87.8 ± 12.1
Time to stable prosthesis (weeks)	8.1 ± 1.4

**Figure 3:**
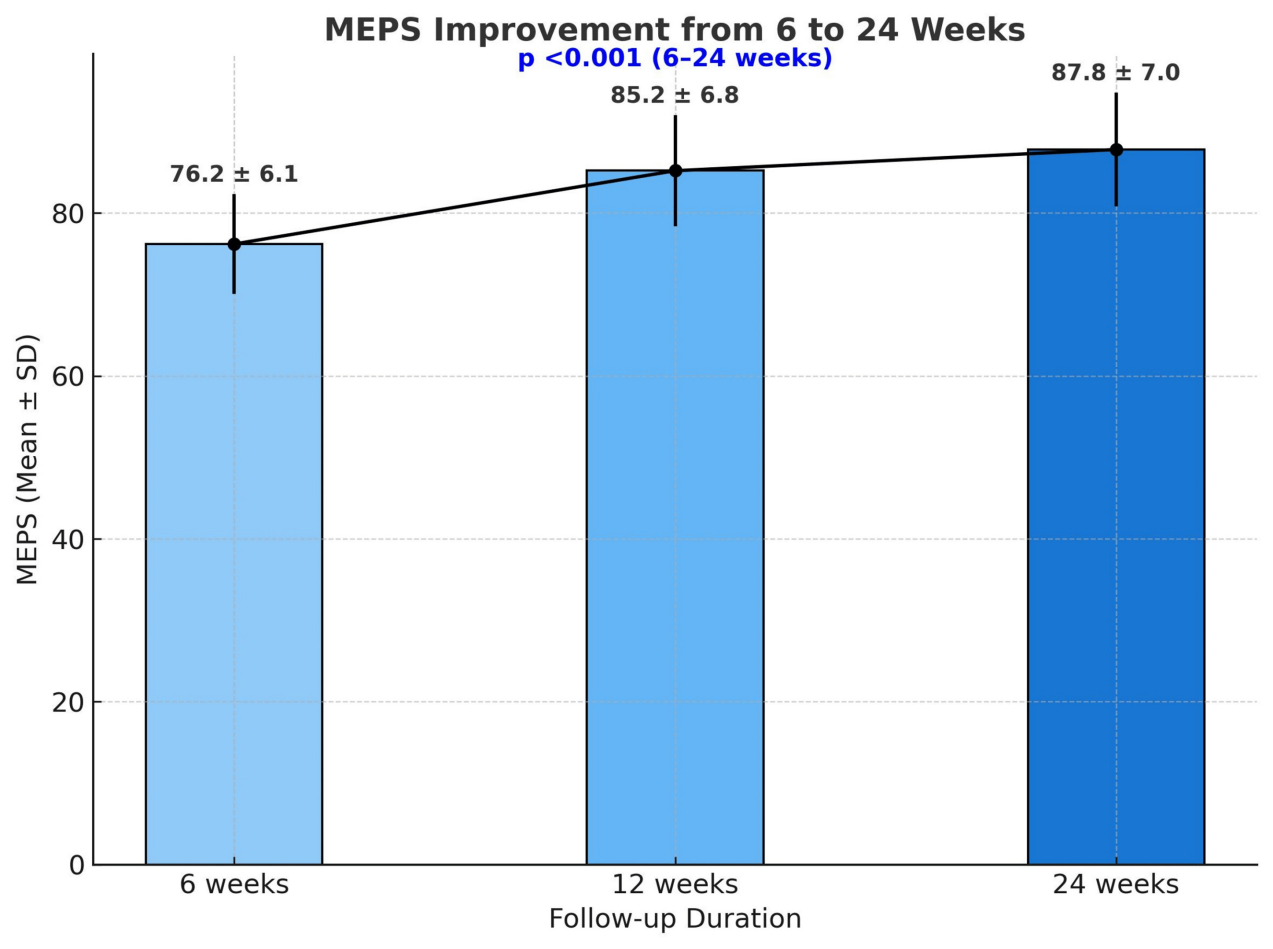
MEPS improvement over a six month period MEPS - Mayo Elbow Performance Score

The following line graph illustrates the improvement in Mayo Elbow Performance Score (MEPS) over the six-month follow-up period (Figure 3).

Complications occurred in eight (32%) of patients. The most frequent complications were infection (n=2, 8%) and stiff elbow (n=2, 8%), followed by unstable elbow (n=1, 4%), heterotopic ossification (n=1, 4%), and implant loosening (n=1, 4%) (Table 3). Infections were superficial, identified at six weeks post-surgery, and resolved with intravenous antibiotics (ceftriaxone, 1 g twice daily for 10 days) without requiring prosthesis removal. The mean flexion-extension arc at the final follow-up was 118° ± 15°, with a mean pronation of 72° ± 10° and supination of 68° ± 12°. The mean total rotational arc (pronation + supination) was 140° ± 18°. Stiff elbow cases, defined as a loss of elbow flexion-extension arc >30° compared with the contralateral side, were observed in two patients with associated injuries (one with an olecranon fracture, one with an elbow dislocation). These patients underwent physical therapy, with one achieving partial improvement (MEPS 74 at 24 weeks) and the other remaining limited (MEPS 58, poor outcome). The single case of unstable elbow occurred in a patient with a type IV fracture and a terrible triad injury, requiring revision surgery to address ligamentous instability. Heterotopic ossification, identified radiologically at 12 weeks, was asymptomatic and did not require intervention. Implant loosening, observed in one patient with a type III fracture at 24 weeks, was associated with a lower MEPS (60) and required ongoing monitoring but no immediate revision. Fisher's exact test showed no significant association between complication rates and Mason classification (p=0.275), likely due to the small sample size of type IV fractures.

**Table 3:** Frequency of complications

Complication	Number of patients (n=25)	Percentage (%)
Infection	2	8.00%
Stiff elbow	2	8.00%
Unstable elbow	1	4.00%
Heterotopic ossification	1	4.00%
Implant loosening	1	4.00%
None	17	68.00%

In independent t-test comparing the Mayo Elbow Performance Score (MEPS) at 24 weeks between patients with Mason type III (n=21, 84%) and type IV (n=4, 16%) radial head fractures, the mean MEPS for type III fractures were 87.1 ± 12.8, indicating good functional outcomes, while Type IV fractures had a slightly higher mean MEPS of 91.3 ± 7.5, suggesting excellent outcomes. The t-statistic (-0.649) and p-value (0.523) indicate no statistically significant difference in functional outcomes between the two fracture types (Table 4). No significant difference in MEPS was observed between Mason type III and type IV fractures at 24 weeks (p=0.523).

**Table 4:** Independent t-test results for MEPS by Mason type MEPS - Mayo Elbow Performance Score

Outcome measure	Mason type III (n=21)	Mason type IV (n=4)	t-statistic	p-value
MEPS (24th Week)	87.1 ± 12.8	91.3 ± 7.5	-0.649	0.523

## Discussion

The management of comminuted radial head fractures, particularly Mason types III and IV, remains challenging due to associated ligamentous instability and limited vascular supply to the radial head, which predisposes to complications such as osteonecrosis and non-union with open reduction and internal fixation (ORIF) [10]. Radial head replacement (RHR) offers a viable alternative by restoring elbow stability and function. This prospective study evaluated functional and radiological outcomes in 25 patients undergoing RHR, demonstrating alignment with existing literature while highlighting favorable early recovery.

In our study, the mean age was 40.2 ± 13.5 years, consistent with studies by Duckworth et al. (2014) and Foroohar et al. (2024), which identified middle-aged adults as the most commonly affected group due to higher activity levels and trauma exposure [11,12]. Males accounted for 64% of cases, corroborating findings by Kovar et al. (2013), who reported a male predominance (∼70%) in radial head fractures, likely due to occupational and recreational risk factors [13]. Right-sided involvement (56%) was more frequent, similar to the 60% prevalence noted in Kaas et al. (2009), possibly due to dominant arm use during falls or trauma [1]. In 21 (84%) patients included in the study, the cause of fracture was a roadside accident. A fall in three cases (12%) and assault in one case (4%) were other causes observed. This correlates with the findings of Mannan et al., where approximately 77% of fractures were due to roadside accidents and 16% fractures were due to falls from height [14]. Whereas research done by Chen et al. showed a mixed pattern of mode of injury 41% by motor vehicle accident, 38% by fall, and others by sports injury or assault [15].

Our study of 25 Mason type III/IV radial head fractures aligned with the seminal work by Ring et al. on 56 similar cases. Both studies confirm the predominance of Mason type III fractures (84% in ours vs. 72% in Ring's cohort). In our study, nine patients (36%) presented with associated injuries, a finding that is consistent with the 30-40% incidence of concomitant injuries reported by Ring et al. in comminuted radial head fractures [6]. The mean MEPS of 87.8 ± 12.1 at six months indicates good to excellent functional recovery in most patients, consistent with findings by Abdulla et al., who reported satisfactory outcomes with pyrocarbon prostheses [9]. The progressive improvement in MEPS from 76.2 at six weeks to 87.8 at 24 weeks (Figure 3) suggests that early mobilization and rehabilitation enhance functional outcomes.

The mean time to stable prosthesis (8.1 ± 1.4 weeks) reflects reliable integration, with only one case of implant loosening. Infection and stiff elbow (8% each) were the most common complications, followed by unstable elbow, heterotopic ossification, and implant loosening (4% each). These rates are lower than those reported by Duckworth et al., who noted higher complication rates in RHR [11].

Compared to ORIF, RHR offers better outcomes for comminuted fractures, as ORIF is associated with higher rates of nonunion and fixation failure [6,7]. Ring et al. reported unsatisfactory outcomes with ORIF for comminuted Mason type III fractures, supporting RHR for such cases [6]. Our findings align with Lópiz et al., who recommended RHR over resection for unstable fractures due to lower complication rates [2].

The lack of significant differences in MEPS between Mason type III and type IV fractures (p=0.523) suggests RHR is equally effective for both fracture types when associated injuries are addressed. The 36% incidence of associated injuries (e.g., olecranon fractures, elbow dislocations) underscores the need for comprehensive preoperative assessment. The small number of type IV fractures (n=4) limited statistical power for subgroup comparisons, but the trend toward slightly higher MEPS in Type IV fractures (91.3 ± 7.5 vs. 87.1 ± 12.8) may reflect effective management of associated injuries.

Limitations of our study include a small and unbalanced sample size between Mason type III and type IV fractures, short follow-up duration, single-center design, subjective outcome measures, and exclusion of complex cases. These factors may have limited the statistical power of subgroup comparisons and the generalizability of the findings. Although all surgeries were performed by experienced surgeons, the use of different surgical approaches (Kocher, Kaplan, and Boyd) introduces potential variability in soft-tissue handling and postoperative function.

## Conclusions

Radial head replacement remains a reliable treatment for unreconstructable Mason type III and IV fractures, particularly when instability or associated injuries are present. Good functional recovery depends not only on the prosthesis itself but also on appropriate postoperative management. Early initiation of physiotherapy is essential to prevent stiffness and restore motion, especially in patients with concomitant soft-tissue or bony injuries. Prompt recognition and treatment of postoperative infections, typically with short-course intravenous antibiotics, help prevent deep or implant-related complications. Overall, outcomes are optimized when surgical reconstruction is combined with structured rehabilitation and vigilant postoperative care. Larger studies with longer follow-up are needed to evaluate long-term implant survival and functional durability.

## References

[1] Kaas L, van Riet RP, Vroemen JP, Eygendaal D (2008). The incidence of associated fractures of the upper limb in fractures of the radial head. Strategies Trauma Limb Reconstr.

[2] Lópiz Y, González A, García-Fernández C, García-Coiradas J, Marco F (2016). Comminuted fractures of the radial head: resection or prosthesis?. Injury.

[3] Mebouinz FN, Kasse A, Habib Sy M (2020). Results of radial head resection after Mason type 3 or 4 fracture of the elbow. Clin Shoulder Elb.

[4] King GJW, Zarzour ZDS, Rath DA, Dunning CE, Patterson SD, Johnson JA (1999). Metallic radial head arthroplasty improves valgus stability of the elbow. Clin Orthop Relat Res.

[5] Mason ML (1954). Some observations on fractures of the head of the radius with a review of one hundred cases. Br J Surg.

[6] Ring D, Quintero J, Jupiter JB (2002). Open reduction and internal fixation of fractures of the radial head. J Bone Joint Surg Am.

[7] Ruan HJ, Fan CY, Liu JJ, Zeng BF (2009). A comparative study of internal fixation and prosthesis replacement for radial head fractures of Mason type III. Int Orthop.

[8] Morrey BF (1993). Functional evaluation of the elbow. The Elbow and Its Disorders.

[9] Abdulla IN, Molony DC, Symes M, Cass B (2015). Radial head replacement with pyrocarbon prosthesis: early clinical results. ANZ J Surg.

[10] Yamaguchi K, Sweet FA, Bindra R, Morrey BF, Gelberman RH (1997). The extraosseous and intraosseous arterial anatomy of the adult elbow. JBJS.

[11] Duckworth AD, Wickramasinghe NR, Clement ND, Court-Brown CM, McQueen MM (2014). Radial head replacement for acute complex fractures: what are the rate and risks factors for revision or removal?. Clin Orthop Relat Res.

[12] Foroohar A, Prentice HA, Burfeind WE, Navarro RA, Mirzayan R, Zeltser DW (2022). Radial head arthroplasty: a descriptive study of 970 patients in an integrated health care system. J Shoulder Elbow Surg.

[13] Kovar FM, Jaindl M, Thalhammer G (2013). Incidence and analysis of radial head and neck fractures. World J Orthop.

[14] Mannan M, Hamid MA, Shrivastava N, Akbar R, Sarwar AR (2024). Functional outcomes of radial head fractures treated with open reduction and internal fixation (ORIF). Cureus.

[15] Chen AC, Cheng YH, Chiu CH (2021). Long-term outcomes of radial head arthroplasty in complex elbow fracture dislocation. J Clin Med.

